# A Machine Learning Approach to Bridge-Damage Detection Using Responses Measured on a Passing Vehicle

**DOI:** 10.3390/s19184035

**Published:** 2019-09-19

**Authors:** Abdollah Malekjafarian, Fatemeh Golpayegani, Callum Moloney, Siobhán Clarke

**Affiliations:** 1 School of Civil Engineering, University College Dublin, Dublin, Ireland; 2 School of Computer Science, University College Dublin, Dublin, Ireland; 3 School of Computer Science and Statistics, Trinity College Dublin, Dublin, Ireland

**Keywords:** drive-by, bridge, damage detection, machine learning, artificial neural network

## Abstract

This paper proposes a new two-stage machine learning approach for bridge damage detection using the responses measured on a passing vehicle. In the first stage, an artificial neural network (ANN) is trained using the vehicle responses measured from multiple passes (training data set) over a healthy bridge. The vehicle acceleration or Discrete Fourier Transform (DFT) spectrum of the acceleration is used. The vehicle response is predicted from its speed for multiple passes (monitoring data set) over the bridge. Root-mean-square error is used to calculate the prediction error, which indicates the differences between the predicted and measured responses for each passage. In the second stage of the proposed method, a damage indicator is defined using a Gaussian process that detects the changes in the distribution of the prediction errors. It is suggested that if the bridge condition is healthy, the distribution of the prediction errors will remain low. A recognizable change in the distribution might indicate a damage in the bridge. The performance of the proposed approach was evaluated using numerical case studies of vehicle–bridge interaction. It was demonstrated that the approach could successfully detect the damage in the presence of road roughness profile and measurement noise, even for low damage levels.

## 1. Introduction

Transportation networks play a key role in supporting economic growth all over the world. Several factors such as technology development, increasing population, and economic growth, have impact on the increasing demand for transportation and personal mobility. Therefore, keeping pace with these changes is an important challenge for transport infrastructure systems, which need to adapt and improve their performance [1]. Bridges are key components of the transport infrastructure that are in constant use and are subjected to deterioration and damage. Therefore, it is necessary to monitor the structural condition of bridges to ensure their reliability and safety. In most bridge-health monitoring techniques, the common practice is to install several sensors at different positions on the structure. It is possible to monitor the structural condition of the bridge by monitoring the responses measured by the sensors. However, depending on the location and type of bridge, in many cases, the direct installations are relatively expensive, time-consuming, and even dangerous. Moreover, covering all bridges within an entire railway or highway network, would require many sensors, power installation for the sensors and an extensive network of data acquisition and processing.

Recently, many researchers have studied ‘indirect’ or ‘drive-by’ methods in which the bridge condition is monitored using an instrumented vehicle [2]. In this method, the vehicle excites the bridge and at the same time measures its response on a moving reference. This is a low-cost approach at the network level as it reduces the need for direct installation of monitoring equipment on the bridge. This method was first proposed by Yang et al. [3] for identifying bridge natural frequencies from responses measured on a passing vehicle. Many numerical and experimental [4,5,6] studies have been carried out aiming at improving the identification of frequencies. Recently, several researchers have investigated estimating bridge mode shapes from indirect measurements. Malekjafarian et al. [7] proposed the short-time frequency domain decomposition (STFDD) method for identifying the bridge mode shapes from the dynamic responses of two following axles. Yang et al. [8] proposed using the Hilbert amplitude of a filtered response measured on a passing axle for finding bridge mode shapes. Malekjafarian et al. [9] suggested using a vehicle equipped by an exciter for finding the bridge mode shapes with higher resolution and better accuracy. Bridge mode shapes identified from indirect methods can be used for bridge damage detection [10,11]. The bridge modal properties can be used to identify the bridge damage, as they lead to changes in these properties. A common challenge in most modal-based approaches is the limited measurement time. This results in a relatively short-time measured signal, when the vehicle travels at a normal highway speed over a short or medium span bridge. Reducing the speed of the test vehicle in highways is also not recommended as it might cause congestion [2].

There are several indirect methods for bridge condition monitoring that do not explicitly estimate bridge modal properties. O’Brien et al. [12] employ a Moving Force Identification (MFI) algorithm to monitor the bridge condition using vehicle accelerations. In their method, road surface profile and global bridge stiffness are calculated using the vehicle–bridge interaction force, assuming the presence of a priori information about dynamic properties of the vehicle (e.g., suspension stiffness, damping, etc.). Li et al. [13] proposed a multistage damage detection technique incorporating empirical mode decomposition (EMD) and a Genetic Algorithm (GA) approach. Quirke et al. [14] employed an optimization method to calculate the apparent profile from simulated acceleration responses of a train bogie. They showed that the calculated apparent profile is sensitive to bridge damage. Zhu et al. [15] proposed using the vehicle–bridge interaction force estimated from the accelerations measured at both the axle and the body for bridge damage detection. They concluded that the interaction force along the travelling pass of the vehicle was more sensitive to bridge damage than the vehicle accelerations.

Despite all the progress that has been made so far, there are still several barriers for indirect methods to become practical [16]. It is generally accepted that the change in bridge natural frequencies due to damage is small and hard to detect. In addition, frequencies might change by other factors such as environmental and operational conditions (e.g., temperature change). The bridge mode shapes from indirect methods are usually identified under certain assumptions (e.g., low vehicle speed, smooth road surface profile, etc.) and need more accuracy to be employed as a damage indicator. The effect of road surface profile is one of the main challenges. A rough profile normally excites vehicle-related frequencies, which might mask bridge-related components of responses. The concept of subtracting signals from identical axles [17] has been used in many studies to address this problem. In addition, the interference from environmental effects such as temperature has never been studied for indirect approaches. As most indirect approaches focus on damage indicators obtained from single runs from healthy and damaged cases, the effects of road profile and temperature change would be significantly misleading in the process of damage detection. The use of multiple runs is a promising approach to tackle these issues. A few studies have been done on using multiple vehicle runs for indirect bridge-damage detection. Cerda et al. [18] employed 60 vehicle runs over a bridge in a laboratory-scaled experimental set up. They developed a detection procedure to capture the shifts in the fundamental frequency of the bridge, due to damage. Miyamoto et al. [19] proposed a framework for monitoring short- and medium-span bridges on a regular basis, in a particular area, using a fixed-route in-service public bus. They employed average-estimated (characteristic) deflections from multiple runs as a damage indicator. A common challenge in most of these methods is how to process large quantities of measured data and transform it into helpful information about the bridge’s condition.

In recent years, there has been an increasing interest in the use of machine learning algorithms for bridge-structural-health monitoring using direct measurements. The algorithms have been employed to extract structural abnormalities from the measured data. These algorithms build and train a model using the data measured from the normal conditions of a structure [20]. For example, Jin et al. [21] proposed an extended Kalman filter-based artificial neural network (ANN) for bridge-damage detection under temperature changes. One-year measurement data from a real bridge was used to train the neural network. Diez et al. [22] proposed a clustering-based machine learning approach, which separated the behaviors of working in normal conditions from the ones working in abnormal conditions. The effectiveness of the proposed approach was shown using data measured from a field test. Gonzales and Karoumi [23] used the bridge acceleration data and bridge weight-in-motion data from the healthy condition to train an ANN. A damage indicator was developed using the error between the measured bridge accelerations and the ones predicted by their ANN model. The method was further developed by Neves et al. [20] using a three-dimensional (3D) bridge model. They added a simplified method for the calculation of the expected total cost of the proposed strategy, as a function of the chosen threshold.

In this paper, a two-stage indirect bridge-damage detection method was proposed using a machine learning algorithm. In the first stage, an ANN was trained using either vehicle accelerations or its Discrete Fourier Transform (DFT) spectrum and speed from multiple passes over a healthy bridge. The vehicle speed was constant in each pass, but varied randomly from one pass to another. The network predicted the vehicle’s response using the vehicle speed. The prediction error, which was the difference between the predicted and measured accelerations, was calculated for each pass. As the healthy condition was used for training, the prediction errors for the training data set were very small. In the second stage, a Gaussian process was fitted to the prediction errors under healthy conditions. This process could automatically detect any subsequent increase in the prediction error that was caused by damage. A numerical model of a vehicle–bridge interaction (VBI) was used to evaluate the effectiveness of the proposed method. Several data sets measured from the healthy and damaged cases were employed. It was shown that the proposed algorithm successfully detected the presence of damage. The proposed method was tested with a relatively high vehicle speed, which overcame the speed limitation of indirect methods. To the knowledge of the authors, this was the first time a machine learning method was used for indirect bridge-damage detection.

## 2. Damage Detection Algorithm

**Figure 1:**
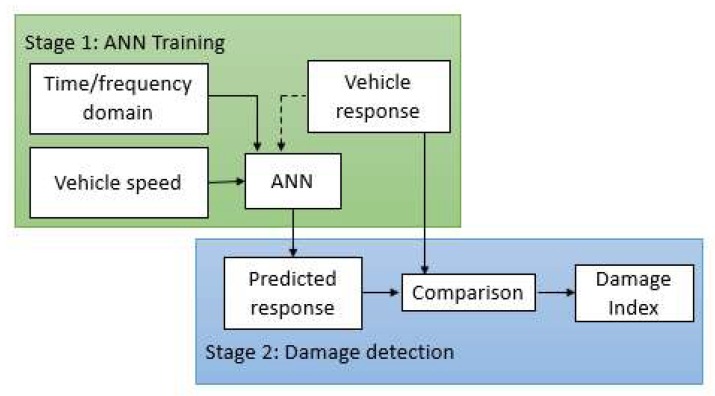
The proposed algorithm.

The proposed damage detection method consists of two stages (Figure 1). An ANN is trained in the first stage to predict vehicle response (accelerations in the time domain or DFT spectrum in the frequency domain). The second stage compares the predicted and measured responses using a Gaussian process. This process classifies the data into healthy and damaged signals based on the prediction error. A brief background of ANN and the details of each stage are described in the following sections.

### 2.1. ANN Background

**Figure 2:**
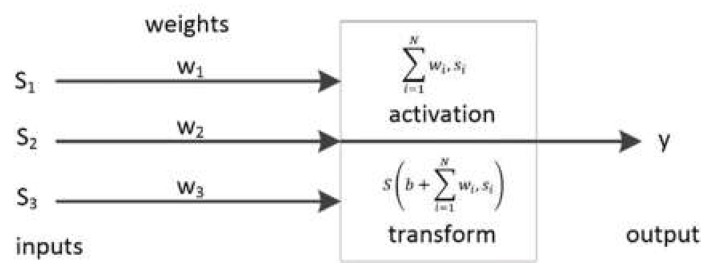
Neurons output calculation.

ANN is a useful tool for predicting/estimating one or multiple output targets in complex systems, where output targets are dependent on multiple input parameters. It has been used in many cases for solving non-linear prediction problems, pattern recognition, and optimization. ANN’s design includes an input layer, hidden layer(s), an output layer, connection weights and bias, an activation function, and a summation node. Each layer integrates a number of computational units called neurons [24], which takes its input values from the previous layer and generates an output value for the next layer. The input layer provides the input values of the network, which are fed to the hidden layer. Each hidden layer consists of a number of neurons that calculate an output using all inputs of the input layer and a predefined set of weights and bias, as stated. This result can be either fed to a next hidden layer or the output layer. The output layer then analyses all the input produced in the last hidden layer and produces the final output of the whole ANN. As depicted in Figure 2, during the learning process, each neuron calculates a single output value based on its input data from the previous layer. In this figure, $s_{i}$ is the output of the neuron $n_{i}$ in the previous layer, $w_{i}$ is a real-valued weight factor associated with $n_{i}$, b is a real number called the neuron bias, and S is some transform function, typically a sigmoid.

A supervised learning technique is one of the methods used to train an ANN network, which is based on the difference between predicted values and the real expected output values. One of such methods is the Levenberg–Marquardt backpropagation [23]. The learning process in an ANN works as a closed loop where the calculated error is the feedback signal to the system and this loop ends when the difference between the predicted values and the measured values are minimized. To do so, the process has to reach a state of $\tau^{*}=\;\tau$, where $\tau^{*}$ is the optimized vector of parameters of the ANN function and τ is a vector of parameter for the ANN. The number of parameters to determine depends on the number of neurons, at each layer of the network. As stated in Equation (1), for a pair of data points $\left(s_{i},y_{i}\right)$, a $\tau^{*}\;$, the optimized vector of parameters of the ANN function minimizes the value predicted by the neural network using the input data, and the actual expected value is:

(1) $$\tau^{*}=\arg\left\{\left|ANN\left(\tau ,s_{i}\right)-y_{i}\right|\right\}$$

In this paper, the hidden layers of the ANN contain hyperbolic tangent (TanH) activation functions and a linear activation function is used in the output layer. The network is designed using a backpropagation optimization method. Each hidden layer has a set number of neurons, each of which contain a transformative function and are assigned an adjusted weight, based on the resulting error from each iteration. The number of hidden layers and neurons should be chosen such that the network maintains the right balance between increased accuracy and required computational time. Initially to train the network, the neurons are assigned random small influence weights and the inputs are propagated through the hidden layers to give a predicted output value. The error between the target output and network predicted output is found. The training error is back-propagated through the layers and the weights are adjusted accordingly. A loop is formed and the process of back-propagating the error of each iteration is repeated until a minimum error is achieved or the predicted and target outputs converge. The backpropagation method employed in this network is the Levenberg–Marquardt backpropagation (LMBP) algorithm. LMBP is known for having a good computation time while also maintaining a stable convergence. The LMBP algorithm combines the favourable aspects of the steepest-descent method and the Gauss–Newton method. The steepest-descent method contributes to the analytical stability of the method while the computational speed of this method can be attributed to the Gauss–Newton method. In this study, the ANN includes two hidden layers and twenty neurons. This model will be used in the numerical examples in this paper.

### 2.2. The Proposed ANN Model

In the proposed algorithm, ANN is trained to predict the vehicle response during its passage over the bridge, where the vehicle speed is known.

The algorithm is proposed to work with two types of responses; raw acceleration signals in the time domain and also the DFT spectrum of the acceleration in the frequency domain. In this study, Fast Fourier Transform (FFT) was used to compute the DFT spectrum. The algorithm proposed here is named acceleration-based or FFT-based algorithms on the basis of which the response type was used. In both versions, it was assumed that there is no other vehicle on the bridge when the test vehicle is crossing the bridge. This might limit the application of the proposed method, but this is likely to happen for small and medium-span bridges.

#### 2.2.1. Acceleration-Based Algorithm

As the sensor measures the response at a moving reference, the location of the vehicle is also known and is used as another input. In this form, the vehicle acceleration is function of the vehicle position and speed for each individual bridge [25]. The independent variable s is formed by the vector containing:

(2) $$s_{i}=\left(l_{i},v_{i}\right)$$

where $l_{i}$ is the position of the vehicle over the bridge at sample *i* and $v_{i}$ is the vehicle speed which is kept constant for each pass. The dependent variable $y_{i}$ can be formed as:

(3) $$y_{i}={\ddot{u}}_{v}{}_{i}$$

where ${\ddot{u}}_{v}{}_{i}$ is the vehicle acceleration at sample *i*.

#### 2.2.2. Spectrum-Based Algorithm

The second version of the algorithm works with the FFT spectrum of acceleration. The frequency domain is defined using the scanning frequency of the measurement. However, a range of frequency that is closer to the bridge frequencies (e.g., 0–20 Hz) can be chosen. In this form, the amplitude of the vehicle acceleration’s spectrum is a function of the frequency and vehicle speed for each individual bridge. The independent variable s is defined by a vector containing:

(4) $$s_{i}=\left(f_{i},v_{i}\right)$$

where $f_{i}$ is the corresponding frequency at sample *i* and $v_{i}$ is the vehicle speed which is kept constant for each pass. The dependent variable $y_{i}$ can be formed as:

(5) $$y_{i}=a_{{\ddot{u}}_{i}}$$

where $a_{{\ddot{u}}_{i}}$ is the amplitude of the FFT spectrum of the vehicle acceleration at sample *i*.

### 2.3. Damage Indicator

The prediction error of the ANN model is characterised by root mean square, calculating the difference between the predicted values and the actual values for each entry point of the data:

(6) $$pe_{j}=\sqrt{\frac{1}{n}\overset{n}{\underset{i=i}{\sum }}{\left(ANN\left(\tau^{*},s_{i}\right)-y_{i}\right)}^{2}}$$

where $pe_{j}$ is the prediction error for passage, j, of the vehicle over the bridge, which is the summation of prediction error for all the data samples corresponding to j.

It is expected that $pe_{j}$ varies significantly for a healthy structure with different vehicle speed. This results in a stochastic distribution that requires a normal distribution characterization to identify the unhealthy and healthy structures. In this paper, a Gaussian process is employed to generate a normal distribution of the prediction errors with mean, μ, and standard deviation, σ values:

(7) $$pe_{j}\sim Ν\left(\mu ,\sigma \right)$$

The prediction errors for the training data set from a healthy bridge would be fairly low. The errors would remain low for a new data set, while the structure is in a healthy condition. A damage index (DI) is introduced using the sum of the distance of the $pe_{j}$ to the mean measured in standard deviations, thus:

(8) $$DI_{j}=\frac{pe_{j}-\mu_{training}}{\sigma_{training}}$$

where $\mu_{training}$ and $\sigma_{training}$ are estimated mean and standard deviation of the prediction errors of the training data set which is from the healthy condition, respectively. Equation (8) is used to standardize the DI. The standardization is helpful for recognizing the healthy condition from low levels of damage. If only $pe_{j}$ is used as a damage indicator, it would show low levels of damage for a healthy case, which is in fact the error of training. Therefore, DI is standardized using $\mu_{training}$ and $\sigma_{training}$. This is shown with more details in the following sections.

## 3. Numerical Modeling

The proposed method is tested using a numerical model of a VBI. This section provides details of the numerical model using finite element (FE) and also numerical modeling of damage on the bridge.

### 3.1. Finite Element Modeling of Vehicle–Bridge Interaction

**Figure 3:**
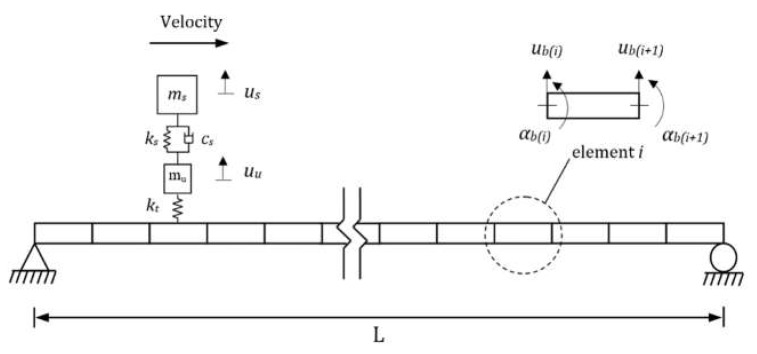
Finite element (FE) model of a quarter-car passing over a bridge.

The VBI, modeled here using FE, works as a coupled VBI system and the solution is calculated at each time step using an iterative procedure. The vehicle is modeled as a quarter-car shown in Figure 3. This model has been extensively used in the literature [25,26,27] as it illustrates many of the important characteristics of VBI [28]. The quarter-car has two independent degrees of freedom corresponding to body mass and axle mass translations. *m_s_* and *m_u_* represent the vehicle body and axle component masses, *u_s_* and *u_u_* represent their displacements, respectively. A spring with linear stiffness *k_t_*, which represents a tyre, connects the axle mass to the road surface. The equations of motion of the vehicle model are obtained in terms of the degrees of freedom by imposing equilibrium of all forces and moments acting on the vehicle:

(9) $$M_{v}{\ddot{u}}_{v}+C_{v}u_{v}+K_{v}u_{v}=f_{int}$$

where $M_{v}$, $C_{v},$ and $K_{v}$ are the respective mass, damping, and stiffness matrices of the vehicle and ${\ddot{u}}_{v}$, ${\dot{u}}_{v}$, and $u_{v}$ are the respective vectors of nodal acceleration, velocity, and displacement ($u_{v}={\left[\begin{array}{cc} u_{s} & u_{u} \end{array}\right]}^{T}$). The vector $f_{int}$ contains the time-varying dynamic interaction forces applied to the vehicle’s degrees of freedom. The interaction forces are function of the road irregularities and bridge vibrations. Therefore, these forces transfer the bridge vibrations to the vehicle as an excitation. It means that the bridge’s natural frequencies would present in the vehicle response which proposes the main idea of indirect bridge monitoring.

Beam finite elements are used to model the bridge as a simply supported beam with total span length *L* (Figure 3). Each beam element consists of four degrees of freedom (one translational and one rotational for each of the two nodes). It has constant mass per unit length, *m*, modulus of elasticity, *E*, and second moment of area, *J*. The equations of motion of the beam under a series of moving time-varying forces can be written in terms of its degrees of freedom:

(10) $$M_{b}{\ddot{u}}_{b}+C_{b}u_{b}+K_{b}u_{b}=f_{int}$$

where $M_{b}$, $C_{b},$ and $K_{b}$ are the global mass, damping and stiffness matrices of the beam model, respectively, and ${\ddot{u}}_{b}$, ${\dot{u}}_{b},$ and $u_{b}$ are the vectors of the nodal bridge accelerations, velocities, and translation, respectively. Rayleigh damping is adopted to represent viscous damping for the bridge [29]:

(11) $$C_{b}=\beta_{1}M_{b}+\beta_{2}K_{b}$$

where $\beta_{1}$ and $\beta_{2}$ are constants. The damping ξ is assumed to be proportional for all modes and $\beta_{1}$ and $\beta_{2}$ are obtained from, $\beta_{1}=2\xi \omega_{1}\omega_{2}/\left(\omega_{1}+\omega_{2}\right)$ and $\beta_{2}=2\xi /\left(\omega_{1}+\omega_{2}\right)$ where $\omega_{1}$ and $\omega_{2}$ are the first two natural frequencies of the bridge [29]. The dynamic interaction between the vehicle and the bridge is implemented in MATLAB. The vehicle and the bridge are coupled at the tyre contact points via the interaction force vector. Combining Equations (9) and (10), the coupled equation of motion of the vehicle and the bridge is formed as:

(12) $$M_{g}\ddot{u}+C_{g}\dot{u}+K_{g}u=F$$

where $M_{g}$ and $C_{g}$ are the combined system mass and damping matrices, respectively, $K_{g}$ is the coupled time-varying system stiffness matrix and *F* is the system force vector. The vector, $u={\left\{u_{v},u_{b}\right\}}^{T}$, is the displacement vector of the system. The Wilson–Theta integration scheme [30] is used to solve the equations for the coupled system with the optimal value of the parameter θ = 1.420815 for unconditional stability in the integration scheme. The initial condition of the solution is considered to be zero vertical translation, velocity, and acceleration in all simulations.

A quarter-car with the properties given in Table 1 is modelled to pass over a bridge. The bridge is modeled using 20 elements with the properties given in Table 2.

### 3.2. Damage Modeling

The damage is modeled by imposing a crack at a particular element of the bridge using the crack modeling method proposed by Sinha et al. [31]. Accordingly, the crack is deemed to cause a stiffness loss in a region on each side of it, with the flexibility varying linearly on each side from the uncracked to the cracked beam section. The flexural rigidity close to the crack, $EI_{e}\left(\zeta \right)$, is given by:

(13) $$EI_{e}\left(x\right)=\{\begin{array}{c} EI_{0}-E\left(I_{0}-I_{c}\right)\frac{\left(x-\zeta_{1}\right)}{\left(\zeta_{c}-\zeta_{1}\right)}\;if\;\zeta_{1}\ll x\ll \zeta_{c}\;\\ EI_{0}-E\left(I_{0}-I_{c}\right)\frac{\left(\zeta_{2}-x\right)}{\left(\zeta_{2}-\zeta_{c}\right)}\;if\;\zeta_{c}\ll x\ll \zeta_{2} \end{array}$$

where $\zeta_{c}$ is the location of the crack and $\zeta_{1}$and $\zeta_{2}$ are positions on either sides of crack where the stiffness reduction begins, $I_{0}$ and $I_{c}$ are the second moment of areas of the undamaged beam and at the crack location, respectively.

**Figure 4:**
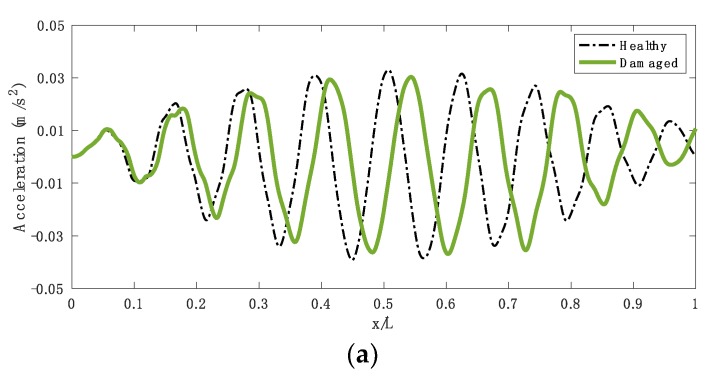

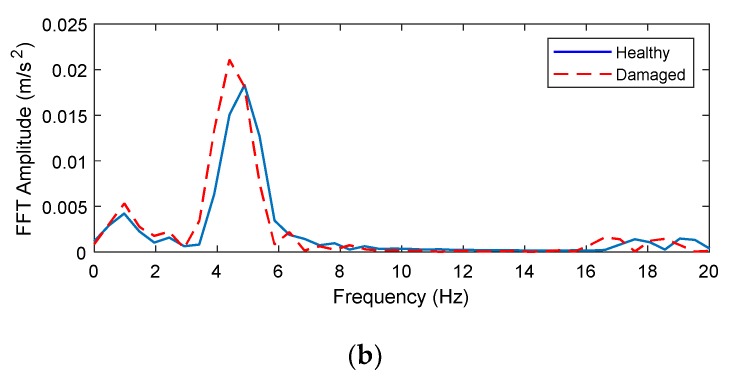
The response measured on the axle of the vehicle passing over healthy and damaged bridges (*x/L* = *vt/L*) (**a**) acceleration and (**b**) FFT spectrum. Reproduced from *OBrien et al. Application of empirical mode decomposition to drive-by bridge damage detection.* *Eur. J. Mech. A Solid 2017; 61, 151–163.* Copyright © *2017* Elsevier Masson SAS. All rights reserved [25].

The severity of the damage is represented by the crack depth, expressed as a ratio of the beam depth. Figure 4 shows the acceleration responses measured on the axle of the vehicle passing over healthy and damaged bridges, where there is a crack with a ratio of 0.3 at the seventh element in the damaged case and a smooth road roughness is considered. Several crack dimensions are considered in the following sections, in a range between 0.05 to 0.3 crack ratios, which is referred to as 5% to 30% damage.

## 4. Numerical Results of the Acceleration-Based Algorithm

### 4.1. A Moving Quarter-Car Passing Over a Bridge with Smooth Road Profile

The numerical VBI model explained in Section 3 is used here to create the data sets. The vehicle is simulated to pass over the healthy bridge, 100 times, at a speed randomly chosen between 10 to 15 m/s for the training (training data set). The number of measurements is chosen to be similar to [23]. No noise is included in the measurements in this section. The ANN model introduced in Section 2.1 is used in this section to predict the response. Once the ANN is trained, seven more batches of 100 passes are simulated including one more over the healthy bridge and another six over the bridge with six damage scenarios (monitoring data set). For the damaged bridge, six levels of damage are considered at the seventh element of the bridge, with cracks with ratios from 0.05 to 0.3, in increment of 0.05.

**Figure 5:**
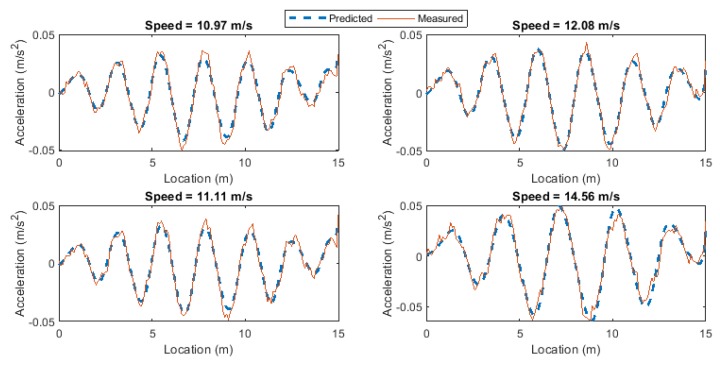
The comparison of the predicted and measured vehicle responses for the first four passes over the healthy bridge.

The ANN is used to predict the vehicle accelerations for seven data sets. The signal is sampled in the space domain at a rate of d*x* = 0.01 m. Therefore, for a total length of 15 m, 1500 samples are recorded for each pass. Figure 5 compares the measured and predicted vehicle accelerations for the first four passes over the healthy bridge, from the monitoring data set. It shows that the signal is predicted with high accuracy.

**Figure 6:**
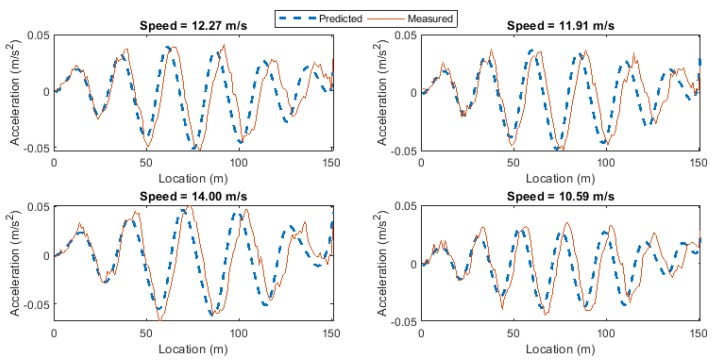
The comparison of the predicted and measured vehicle responses for the first four passes over the damaged bridge with crack ratio of 0.3.

Figure 6 shows the comparison of the measured and predicted vehicle accelerations for the first four passes over the damaged bridge when the crack ratio is 0.3. It can be seen that the ANN predicts the signal with some inaccuracies. When the bridge is damaged, the dynamic behavior of the structure changes. This introduces some changes in the VBI which changes the vehicle response. As the ANN model is only trained on the healthy structure, it cannot accurately predict the vehicle response in presence of damage and the prediction errors increase compared to the healthy condition. Therefore, the prediction error can be used as a damage indicator.

**Figure 7:**
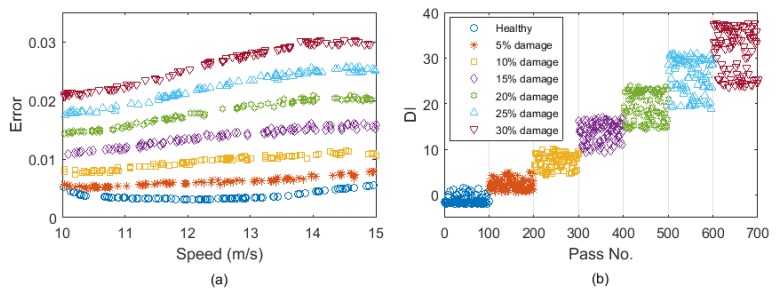
(**a**) The prediction errors and (**b**) DI for the healthy and damaged cases (DI: damage indicator).

Figure 7a shows the prediction errors for the healthy and damaged data cases for all damage scenarios versus the vehicle speed. As the vehicle speed in the new healthy and damaged data sets are different from the training data set, the prediction errors would not be exactly the same for different vehicle passages, even if the bridge condition remains unchanged. Figure 7b shows DI plotted for all passes including the healthy and 6 levels of damage. For the healthy case, the DI values are around zero showing that the bridge condition has not changed. But for the other passes, DI increases as the crack ratio increases. In addition, it is shown in Figure 7a that the prediction error varies significantly with the vehicle speed. However, Figure 7b shows that the Gaussian process used in the DI can separate the change due to the vehicle speed and the change caused by the damage.

### 4.2. Two Moving Quarter-Cars Passing over a Bridge with Low Road Profile

In this section, a road profile is added to the bridge to represent a more realistic case. The irregularities of this profile are randomly generated using the following equation [32]:

(14) $$r\left(x\right)=\underset{i}{\sum }d_{i}\cos\left(n_{i}x_{c}+\gamma_{i}\right)$$

where $n_{i}$ is the *i*th spatial frequency in consideration, ranging from 1 to 100 cycle/m with increment Δn = 0.04 cycle/m, $\gamma_{i}$ is the random phase angle, and $d_{i}$ is the amplitude for each roughness class which is defined by:

(15) $$d_{i}=\sqrt{2G_{d}\left(n_{i}\right)\Delta n}$$

where $G_{d}$ is the displacement Power Spectral Density (PSD) function and is defined as:

(16) $$G_{d}\left(n_{i}\right)=G_{d}\left(n_{0}\right){\left(\frac{n_{i}}{n_{0}}\right)}^{-\omega }$$

where ω=2 and $n_{0}=0.1$ cycle/m and $G_{d}\left(n_{0}\right)$ is determined by the roughness class given in ISO 8608. In this study, $G_{d}\left(n_{0}\right)$ is considered to be 0.01 × 10^−6^ m^3^ which is for a low-surface roughness.

It is shown in previous studies [7,16,25,33] that the presence of a road profile introduces inaccuracies in indirect bridge monitoring methods. In this case, the vehicle frequencies are dominant in the responses measured on the passing vehicle, which might hide the bridge responses. Yang et al. [17] overcame this challenge by proposing the idea of subtracting the responses measured on two following identical axles. It is shown that the bridge frequency is dominant in the residual acceleration response and the effect of the road profile is substantially removed. A similar subtraction idea is employed here.

**Figure 8:**
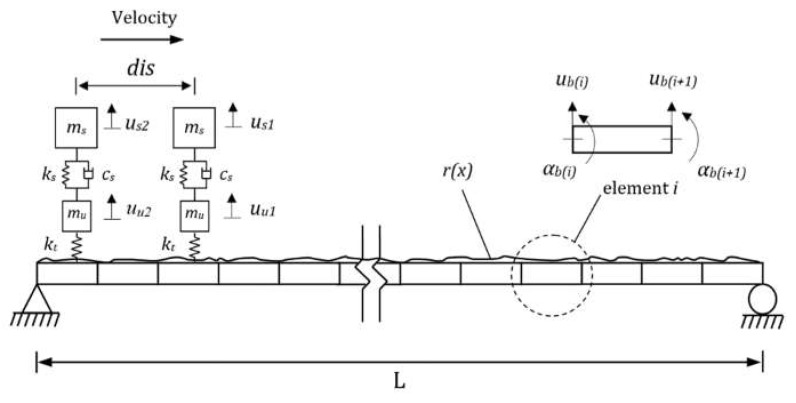
Two quarter-cars passing over the bridge.

Two quarter-cars are modeled to pass over the bridge in this section (Figure 8). The vehicles are assumed to have the same properties. The residual response is obtained by subtracting the acceleration responses of the axles.

(17) $${\ddot{u}}_{res}={\ddot{u}}_{u1}-{\ddot{u}}_{u2}$$

It was shown in [9] that the subtraction idea is sensitive to measurement noise. White noise is added to the calculated axle responses to generate noise-polluted responses:

(18) $${\ddot{u}}_{u}^{polluted}={\ddot{u}}_{u}+E_{P}N_{noise}\sigma \left({\ddot{u}}_{u}\right)$$

where ${\ddot{u}}_{u}^{polluted}$ is the polluted response, $E_{P}$ is the noise level, $N_{noise}$ is a standard normal distribution vector with zero mean value and unit standard deviation, ${\ddot{u}}_{u}$ is the calculated response, and $\sigma \left({\ddot{u}}_{u}\right)$ is its standard deviation. Three levels of noise 1%, 3%, and 5% are added to the axle responses and the residual responses are obtained from the noise-polluted signals.

**Figure 9:**
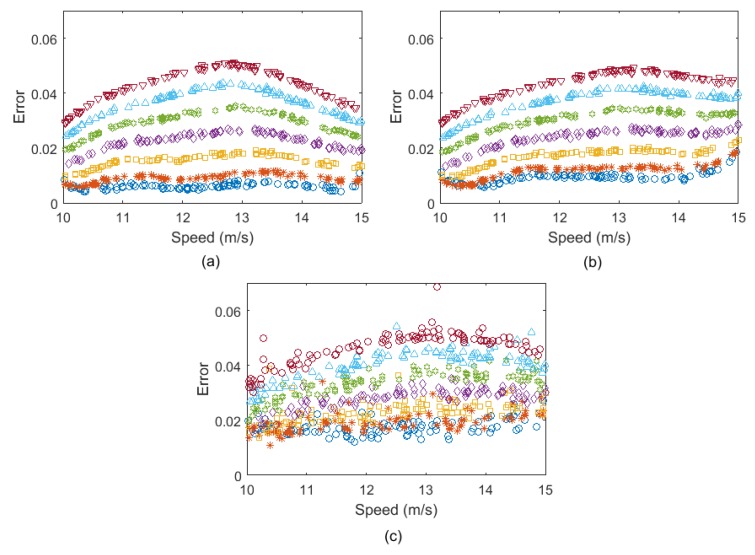
The prediction errors for (**a**) 1% added noise, (**b**) 3% added noise, and (**c**) 5% added noise (dark blue: healthy, red: 5% damage, yellow: 10% damage, purple: 15% damage, green: 20% damage, light blue: 25% damage, brown: 30% damage).

For each noise level, a batch of 100 vehicle passes over the healthy bridge were simulated for training the ANN. The ANN model introduced in Section 2.1 was used in this section to predict the response. Another 7 batches of 100 passes were also generated, one with a healthy condition and the other 6 with different damage levels. The errors calculated for the noise level 1%, 3%, and 5% are shown in Figure 9a–c, respectively. It can be seen that all three noisy cases provide similar levels of errors, but as the noise level increases, the errors for each damage case are less consistent. For the lower noise levels of 1% and 3%, the trends of errors for various damage levels in Figure 9a,b are well separated. Although the errors are not well separated for 5% noise in Figure 9c, but the differences in the distributions of errors are still recognizable.

**Figure 10:**
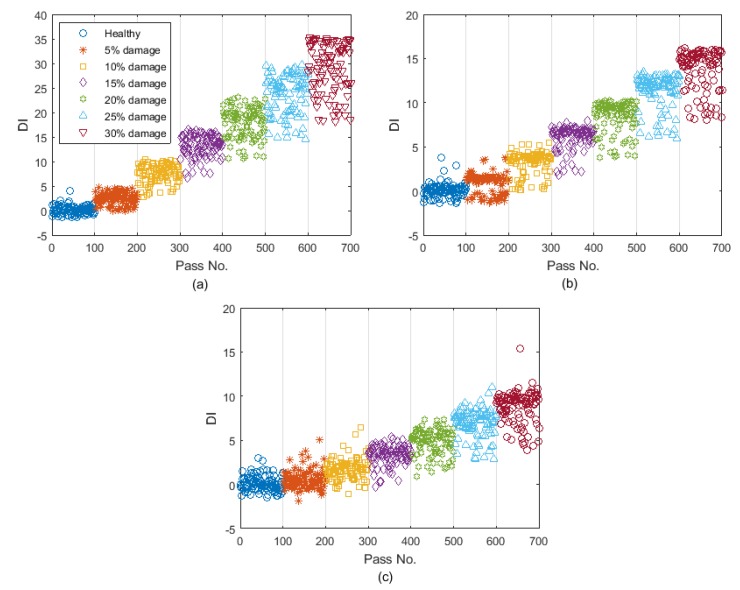
The DIs for (**a**) 1% added noise; (**b**) 3% added noise; and (**c**) 5% added noise.

Figure 10 shows the DI values calculated for the errors in Figure 9. Figure 10a shows that DI provides a relatively high sensitivity to damage as its maximum value is about 35 when 30% damage exists. This sensitivity is reduced by increasing the noise level. The maximum value of DI for 3% and 5% noise is about 15, which is less than half of it for 1% noise. It can be concluded that DI provides lower values for 5% noise compared to 1% and 3% noise levels, meaning that introducing noise to the measurements reduces the sensitivity of the proposed DI to damage. However, most importantly, the proposed algorithm works well even for 5% noise, where there is a clear pattern for DI when the damage level is increased (see Figure 10c).

The acceleration-based version of the proposed method directly works with the signals instead of the bridge modal properties identified from the signals. Therefore, it excludes the inaccuracies that might exist in the identification process. In addition, the time series include all the dynamic properties of the bridge such as natural frequencies and mode shapes, which are taken into account in the proposed method for damage detection. However, in the presence of road profile, it only works with the subtraction of the responses from the following axles, which needs high accuracy measurements that might limit its real-world application.

## 5. Numerical Results of the FFT-Based Algorithm

### 5.1. A Moving Quarter-Car Passing over a Bridge with a Smooth Road Profile

**Figure 11:**
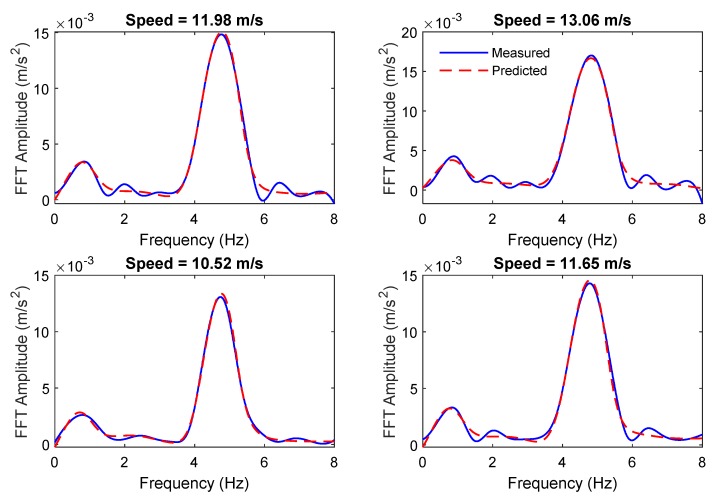
The comparison of the predicted and measured vehicle FFT responses over the healthy bridge.

The data generated in Section 4.1 is used in this section. Instead of prediction acceleration, the ANN is used to predict the FFT spectrum for seven data sets. The ANN model introduced in Section 2.1 was used in this section. The ANN was trained using the training data set including 100 passes over the healthy bridge. Figure 11 compares the measured and predicted vehicle acceleration FFT spectrum for two sample passes over the healthy bridge from the monitoring data set. The results showed that the signal was predicted with a higher accuracy.

**Figure 12:**
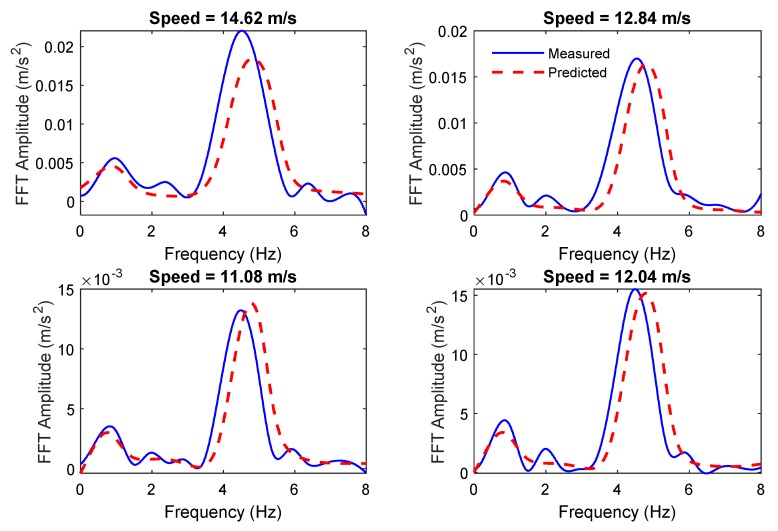
The comparison of the predicted and measured vehicle FFT responses over the damaged bridge with a crack ratio of 0.3.

Figure 12 shows the comparison of the measured and predicted vehicle acceleration FFT spectrums for two sample passes over the damaged bridge when the crack ratio is 0.3. Similar to the acceleration-based algorithm, there were some inaccuracies in the prediction of the FFT spectrum when there was a damage in the bridge.

**Figure 13:**
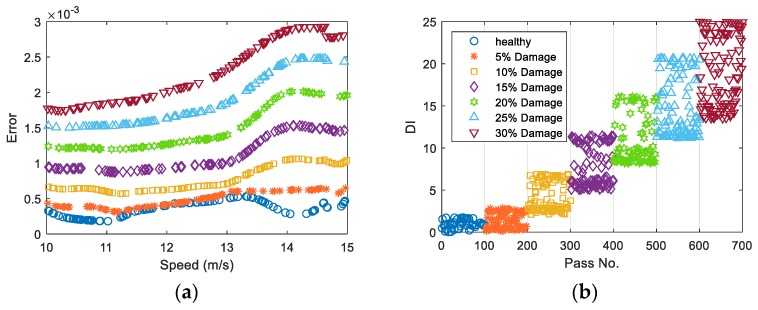
(**a**) The prediction errors and (**b**) the DI for the healthy and damaged bridges.

Figure 13a shows the prediction errors for the healthy and damaged data sets for all different damage levels versus the vehicle speed. Figure 7b shows the DI plotted for all passes including a pass over a healthy bridge and 6 other passes over a damaged bridge, with different levels of damage. Similar to the acceleration-based case, for the healthy bridge, the DI values were around zero showing that the bridge condition had not changed. However, for the other passes, DI increased as the crack ratio increased.

### 5.2. A Moving Quarter-Car Passing over a Bridge with a Low Road Profile

**Figure 14:**
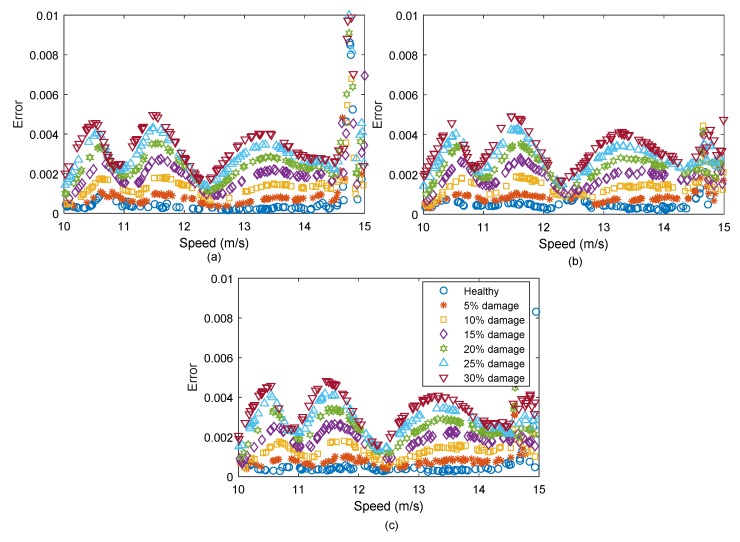
The prediction errors for (**a**) 1% added noise, (**b**) 3% added noise, and (**c**) 5% added noise.

The same road profile used in Section 4.2 was included in the bridge model in this section. One of the main advantages of the FFT-based algorithm over the acceleration-based one is that there is no need to use the residual response from subtraction of the responses obtained from the two following quarter-cars. Therefore, the ANN was trained using the range of 0–8 Hz. Similar to Section 4.2, three levels of noise 1%, 3%, and 5% were added to the axle response and the FFT responses were obtained from the noise-polluted signals. For each noise level, a batch of 100 vehicle passes over the healthy bridge were simulated for training the ANN. The ANN model introduced in Section 2.1 was used in this section to predict the response. Another 7 batches of 100 passes were also generated, one with healthy condition and the other 6 with different damage levels. Figure 14a–c shows the errors calculated for the noise level 1%, 3%, and 5%, respectively.

**Figure 15:**
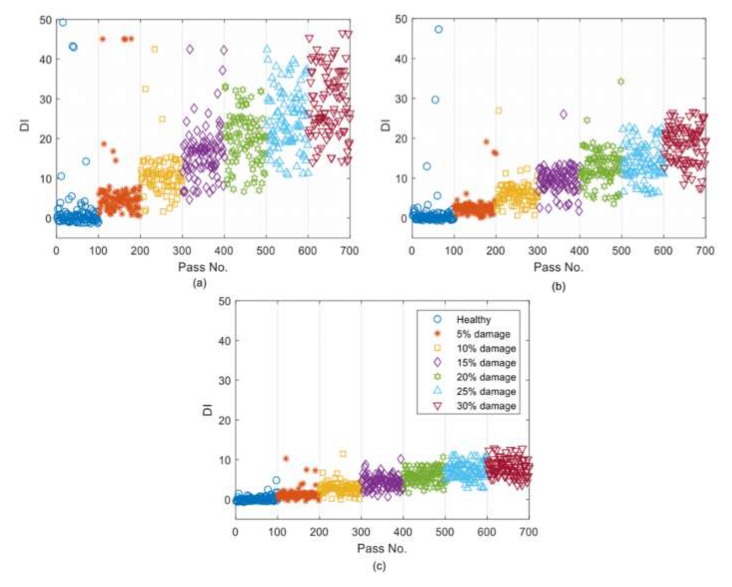
The DIs for (**a**) 1% added noise, (**b**) 3% added noise, and (**c**) 5% added noise.

Figure 15 shows the DI values calculated for the errors in Figure 14. Figure 15a shows that the DI has a relatively high sensitivity to damage as its maximum value is about 50 when 30% damage exists. This sensitivity is reduced by increasing the noise level. The maximum value of DI for 3% and 5% noise is about 30 and 15, respectively. Similar to the acceleration-based algorithm, it can be concluded that introducing noise to the measurements reduces the sensitivity of the proposed DI to the damage.

It is shown that the FFT-based algorithm works well even with the measurements from one quarter-car and does not need the subtraction. This is a great advantage compared to the acceleration-based version of the proposed algorithm. However, it depends on the changes in the FFT spectrum of the signal. Therefore, no information about the damage location can be obtained from the FFT-based algorithm.

## 6. Conclusions

A new bridge-damage detection approach was proposed in this paper using machine learning techniques, combining an Artificial Neural Network model (ANN) and a Gaussian process, to identify healthy bridge condition from unhealthy ones. The ANN model was trained using a training data set including the vehicle responses measured from multiple passes over a healthy bridge. The prediction error for each passage is calculated using root mean square of differences between the measured and the predicted responses. It is shown that distribution of prediction errors would change when the bridge was damaged. To interpret the prediction errors when different vehicle speed was considered, a damage indicator (DI) that was defined using a Gaussian process, was used to normalize the distribution of the prediction errors in presence of different vehicle speed. Several simulated data set with healthy and damaged conditions were simulated. A more complicated case study including a road surface profile and measurement noise was also considered. It was shown that the presence and the level of the damage could be detected using the proposed approach in this condition. However, for real-world applications, in order to ensure the safety of a bridge, a threshold must be defined including the allowed DI value for each bridge. This value could be a function of several parameters, e.g., the location of the damage, the noise level, the input of the ANN (acceleration or FFT), road roughness, etc.

This is one of the earliest attempts using a population of data for indirect bridge health monitoring, which is an important step that needs to be taken for indirect approaches to become practical. For example, the proposed method could detect the change in the dynamic behavior of the bridge. It was assumed that the changes in the structure behavior were due to the damage. However, in real-world applications, other environmental effects (such as temperature change) could also change the behavior. However, this approach also had the potential to be trained for seasonal temperature change to tackle the issue of environmental effects on indirect bridge monitoring. In addition, the method was proposed for detecting the occurrence of the damage and not localizing it. This application was still an important challenge in drive-by methods which are mostly used for monitoring short- and medium-span bridges.

However, there are several challenges that are needed to be addressed for using the proposed method in practice. The number of passes for training and damage detection (chosen to be 100 in this study) needs to be optimized to reduce the computational cost of monitoring. The vehicle speed and position were used as inputs to the ANN model for the acceleration-based algorithm. This means that a high-accuracy positioning system is required to record the position of the vehicle and extract the average speed.

## Figures and Tables

**Table 1:** Properties of the quarter-car. Reproduced from *OBrien et al. Application of empirical mode decomposition to drive-by bridge damage detection. Eur. J. Mech. A Solid 2017; 61, 151–163*. Copyright © *2017* Elsevier Masson SAS. All rights reserved. [25].

Properties	Symbol	Corresponding Value
Vehicle body mass (kg)	*m_s_*	9300
Vehicle axle mass (kg)	*m_u_*	700
Tyre stiffness (N/m)	*k_t_*	1.75 × 10^6^
Suspension damping (Na/m)	*c_s_*	10^4^
Suspension stiffness (N/m)	*k_s_*	4 × 10^5^
Body bounce frequency (Hz)	*ω_b_*	0.94
Axle hop frequency (Hz)	*ω_a_*	8.83

**Table 2:** Properties of the bridge. Reproduced from *OBrien et al. Application of empirical mode decomposition to drive-by bridge damage detection. Eur. J. Mech. A Solid 2017; 61, 151–163*. Copyright © *2017* Elsevier Masson SAS. All rights reserved. [25].

Properties	Symbol	Value
Total length (m)	*L*	15
Depth (m)	*d*	0.75
Second moment of area (m^4^)	*J*	0.5273
Modulus of elasticity (N/mm^2^)	*E*	35,000
Mass per unit length (kg/m)	*m*	28,125
Bridge first natural frequency (Hz)	$\omega_{b}$	5.65
